# Melanoma Presentations Before, During, and After the COVID-19 Pandemic: A Multicenter Cohort Study from North Rhine-Westphalia, Germany

**DOI:** 10.3390/cancers18030539

**Published:** 2026-02-06

**Authors:** Thilo Gambichler, Carmen Colo, Sera Selina Weyer-Fahlbusch, Laura Susok, Stefanie Boms, Nessr Abu Rached

**Affiliations:** 1 Department of Dermatology, Dortmund Hospital gGmbH, University Witten/Herdecke, 44137 Dortmund, Germany; seraselina.weyer-fahlbusch@klinikumdo.de (S.S.W.-F.); laura.susok@klinikumdo.de (L.S.); 2 Department of Dermatology, Skin Cancer Center, Ruhr-University Bochum, 44791 Bochum, Germany; carmen.colo@ruhr-uni-bochum.de (C.C.); nessr.aburached@ruhr-uni-bochum.de (N.A.R.); 3 Department of Dermatology, Skin Cancer Center, Christian Hospital Unna, 59423 Unna, Germany; s.boms@hospitalverbund.de

**Keywords:** SARS-CoV-2, skin cancer, coronavirus, tumor thickness, tumor diameter, volume

## Abstract

Background: The COVID-19 pandemic disrupted access to routine dermatologic care and may have delayed melanoma diagnosis and management. Evidence on the post-pandemic period and on hospital-based referral cohorts remains limited. We assessed melanoma presentations before, during and after the pandemic in three skin cancer centers in North Rhine-Westphalia, Germany. Methods: We conducted a multicenter retrospective cohort study of inpatients with cutaneous melanoma grouped into Phase 1 (February 2017–February 2020), Phase 2 (March 2020–March 2023), and Phase 3 (April 2023–May 2024). The primary endpoint was Breslow tumor thickness (TT) among invasive melanomas, analyzed using multivariable log-linear regression adjusted for center, age, sex, anatomic site, and histologic subtype. Secondary endpoints included T category and AJCC stage distributions (including stage 0/Tis), macroscopic primary tumor specimen dimensions (area and volume; available cases), staging work-up and sentinel lymph node biopsy (SLNB) indicators, and exploratory laboratory parameters (LDH, S100, CRP) and dermal mitotic rate. Results: We included 2960 patients (Phase 1: 1162; Phase 2: 1251; Phase 3: 547). Median TT among invasive melanomas was 1.1 mm (IQR 0.6–2.3), 1.1 mm (0.5–2.4), and 1.0 mm (0.5–2.3) across phases (*p* = 0.037). In adjusted models among invasive tumors, TT did not increase (Phase 2 vs. Phase 1: 0.97, 95% CI 0.90–1.04; Phase 3 vs. Phase 1: 0.94, 0.86–1.03). AJCC stage 0 decreased from 7.7% and 6.1% to 2.0%; adjusted OR Phase 3 vs. Phase 1: 0.24 (95% CI 0.13–0.46). Within invasive tumors, the distribution of T categories (T1a–T4) and AJCC stages I–IV was similar across periods. Among cases with available macroscopic primary tumor specimen dimensions, median area and volume were higher during and after the pandemic (area *p* = 0.030; volume *p* = 0.042), but period effects attenuated in models adjusted for TT. Exploratory analyses suggested a higher proportion of elevated LDH and a lower proportion of elevated S100 across periods, while CRP and dermal mitotic rate showed no clear period shift. Conclusions: In this large melanoma inpatient cohort, the pandemic period was not associated with thicker invasive melanomas after covariate adjustment. However, a persistent reduction in stage 0/Tis presentations in the post-pandemic period suggests ongoing disruption or shifting of early detection and referral pathways. Exploratory increases in macroscopic tumor dimensions may point to changes not captured by thickness alone, but require cautious interpretation given missingness and potential documentation effects.

## Simple Summary

The COVID-19 pandemic raised concerns that melanoma might be diagnosed later because many people had reduced access to routine skin checks. We reviewed almost 3000 melanoma inpatients treated at three German skin cancer centers before, during, and after the pandemic to see whether this happened. After adjusting for patient and tumor factors, invasive melanomas were not thicker during or after the pandemic. This suggests that, within hospital-based care, most melanomas were still diagnosed at similar stages as before. However, very early melanomas (stage 0 or melanoma in situ) became much less common, especially after the pandemic. These earliest cases are usually found through screening, so their decline likely reflects ongoing disruption in early detection and referral pathways. In summary, while advanced melanoma cases did not worsen, fewer early-stage diagnoses indicate that some patients may still be missing timely screening and evaluation.

## 1. Introduction

The SARS-CoV-2 pandemic led to substantial changes in healthcare delivery worldwide. Infection-control measures and the reallocation of resources to emergency care reduced access to routine outpatient services, including medical consultations and cancer screening [1,2,3,4]. In addition, fear of SARS-CoV-2 infection and related anxiety likely discouraged some patients from seeking medical attention, particularly for asymptomatic lesions [3,5]. Because melanoma prognosis is closely linked to stage at diagnosis, delays in detection or treatment can have clinically relevant consequences [6]. Given the heterogeneous natural history of melanoma, a diagnostic delay of approximately one year may primarily affect the detection of melanoma in situ and very thin invasive melanomas rather than consistently shifting Breslow tumor thickness (TT) in selected referral cohorts.

During the pandemic, many studies reported fewer melanoma diagnoses and worse prognostic features at presentation, but results have been heterogeneous [5,7,8,9,10,11,12,13,14,15,16,17,18,19,20]. An early systematic review summarizing evidence published up to September 2022 reported an overall decline in melanoma diagnoses with heterogeneous changes in staging and Breslow thickness [7]. In a global systematic review and meta-analysis, Mostafavi Zadeh et al. quantified an overall decline in melanoma diagnoses and reported pooled evidence of modest increases in TT and other adverse pathological parameters [20]. Using a French multicenter clinical database (RIC-Mel), Skowron et al. described pandemic-associated changes in melanoma presentations and emphasized that reductions in early-stage diagnoses may drive apparent shifts in stage distributions [19]. Registry-based analyses also documented disruption and recovery at the population level; for example, using U.S. SEER data through 2021, Kim et al. reported a marked decline in melanoma incidence in 2020 with incomplete recovery by 2021 [18].

Potential delays have also been described within the treatment pathway. Using the U.S. National Cancer Database, Chung et al. reported increased odds of delayed melanoma treatment during the pandemic [17]. Patient behavior likely contributed in parallel: in the German Mela-COVID survey, Teuscher et al. found that many melanoma patients postponed or missed appointments during the pandemic, frequently driven by fear of SARS-CoV-2 infection [5]. Heterogeneity may also be shaped by patient characteristics and geography. In a nationwide Icelandic analysis stratified by sex, age, and TT, Ahmed et al. described a post-pandemic surge of thick melanomas in women older than 50 years of age [16]. In a U.S. hospital-based cohort, Munjal et al. reported that rurality and longer distance to hospital were associated with greater year-to-year increases in mean TT from 2019 to 2020, suggesting that access barriers may disproportionately affect vulnerable subgroups [15].

North Rhine-Westphalia provides an informative regional context because neighboring German skin tumor centers have reported mixed signals. In two dermatology clinics in North Rhine-Westphalia (Wuppertal and Oberhausen), Balakirski et al. observed stable TT and AJCC stage distributions in 2020–2021 compared with 2019, but reported higher proportions of positive sentinel lymph node biopsies [14]. Extending this work into the post-pandemic period, Mrotzek et al. reported that key parameters returned to pre-pandemic levels only in 2023, highlighting the importance of including post-pandemic observations [13].

Against this background, evidence on the post-pandemic period remains limited, and many datasets do not explicitly consider the selection mechanisms of hospital-based referral cohorts. Furthermore, additional size-related descriptors beyond TT are rarely available. We therefore compared melanoma presentations across pre-pandemic, pandemic, and post-pandemic periods in three certified skin cancer centers in North Rhine-Westphalia. The primary objective was to assess period-related differences in TT among invasive melanomas. Secondary objectives were to evaluate T category and AJCC stage distributions (including stage 0), to explore macroscopic primary tumor specimen dimensions (area and volume) as exploratory size descriptors, and to examine potential heterogeneity across centers and potential effect modification by age and sex. Because two-dimensional and diameter-based descriptors have been proposed as complementary size metrics in melanoma [21,22,23,24], we additionally assessed whether macroscopic area showed period-related shifts independent of TT.

## 2. Materials and Methods

### 2.1. Study Design and Setting

We performed a retrospective multicenter cohort study of patients with cutaneous melanoma managed in three OnkoZert-certified skin cancer centers in North Rhine-Westphalia, Germany (Bochum, Dortmund, and Unna). Only patients with an inpatient episode at the participating centers were eligible according to the study protocol. In this manuscript, the term “melanoma presentations” refers to incident melanoma cases captured at the index diagnosis/first inpatient episode at a study center; repeated follow-up visits were not counted as separate presentations. The index date was the date of first diagnosis (ED), defined as the date of hospital admission or, if applicable, the date of the originating pathology report of the primary melanoma.

### 2.2. Study Periods

Patients were assigned to one of three predefined periods: Phase 1 (pre-pandemic) from February 2017 to February 2020, Phase 2 (pandemic) from March 2020 to March 2023, and Phase 3 (post-pandemic) from April 2023 to May 2024.

### 2.3. Data Source and Variables

Data were abstracted from local clinical and pathology documentation and entered into a harmonized database. Key variables included age at diagnosis (years), sex, center, anatomic site, histologic subtype, ulceration status, TT in mm, T category and AJCC stage at diagnosis. Macroscopic primary tumor specimen dimensions (three orthogonal measures in mm, as documented in pathology reports) were available in a subset. Primary tumor specimen volume (mm^3^) was calculated as the product of the three dimensions. Primary tumor specimen area (mm^2^) was calculated as the product of the first two dimensions (length × width) to provide a two-dimensional descriptor less dependent on the third measure. Laboratory parameters (LDH, S100, CRP) and binary indicators for elevation were available in most patients according to local clinical documentation; cutoffs were pre-specified as LDH ≥ 215 U/L, S100 ≥ 0.11 µg/L, and CRP ≥ 5 mg/L. Dermal mitotic rate was recorded as a binary variable (≥1 mitosis/mm^2^ vs. 0). In most patients, the index diagnostic excision of the primary melanoma was performed in community dermatology practices before referral to the participating centers, which typically provided subsequent wide local excision and staging (including sentinel lymph node biopsy where indicated). Therefore, macroscopic primary specimen dimensions primarily reflect outpatient excision and reporting practices of the originating pathology laboratories rather than surgical technique at the study centers.

### 2.4. Outcomes

The primary outcome was TT among invasive melanomas (T1a–T4). Secondary outcomes were the distribution of T categories and AJCC stages across periods, the proportion of in situ melanoma (AJCC stage 0), macroscopic primary tumor specimen dimensions (area and volume; available cases), and staging work-up indicators (lymph node ultrasound and extended staging). Sentinel lymph node biopsy (SLNB) performance and positivity were analyzed descriptively and as secondary endpoints. Laboratory parameters (LDH, S100, CRP) and dermal mitotic rate were assessed as exploratory endpoints.

### 2.5. Statistical Analysis

Statistical analyses were performed using MedCalc^®^ version 22.014 (Ostend, Belgium) and R version 4.5.1. Two-sided *p* values < 0.05 were considered statistically significant. Categorical variables were summarized as counts and percentages and compared across periods using Pearson’s chi-squared test. Continuous variables were summarized using medians and interquartile ranges (IQR) and compared using the Kruskal–Wallis test because of skewed distributions. For the primary endpoint, we fitted a log-linear regression model with log-transformed TT as the dependent variable and study period as the exposure (Phase 1 as reference), adjusting for age, sex, center, grouped anatomic site (head/neck, trunk, upper extremity, lower extremity, other), and histologic subtype. TT is typically right-skewed; log transformation reduces skewness and the influence of extreme values and enables modeling multiplicative (relative) period effects (Figure S1). Exponentiated coefficients are reported as geometric mean ratios (GMR) with 95% confidence intervals (CI); for example, a GMR of 0.94 corresponds to an approximately 6% lower adjusted mean TT compared with the reference period. Ulceration was not included as a covariate in the TT model because it is closely correlated with TT and contributes to AJCC staging; moreover, ulceration prevalence did not differ across study periods (Table 1), making confounding by ulceration unlikely. For stage 0, we fitted a multivariable logistic regression model (stage 0 vs. stages I–IV) adjusted for age, sex, and center. As a sensitivity analysis for potential case-mix effects, we repeated T category comparisons after excluding in situ cases (Tis) and compared AJCC stages after excluding stage 0. Center heterogeneity was tested by adding a period-by-center interaction term in the TT model and by presenting center-stratified period effects.

For macroscopic primary tumor specimen dimensions (area and volume), we compared distributions across periods using the Kruskal–Wallis test among available cases. As exploratory adjusted analyses among invasive melanomas with both TT and the respective size measure available, we fitted log-linear regression models for log(area) and log(volume) including period and the same covariates as in the TT model, additionally adjusting for log-transformed TT; results are presented as geometric mean ratios. Availability of area and volume by period was assessed using chi-squared tests and exploratory logistic regression adjusted for age, sex, and center.

Exploratory laboratory parameters (continuous values and elevated/not elevated indicators) and dermal mitotic rate were compared across periods using Kruskal–Wallis and chi-squared tests, respectively, among available cases. Multivariable logistic regression models for elevated laboratory markers and dermal mitotic rate were fitted among invasive melanomas with available values, adjusted for age, sex, center and log-transformed TT. Pairwise comparisons for area were Bonferroni-adjusted in a sensitivity analysis.

Missing data. Analyses were performed as complete-case analyses for each endpoint. TT was missing only in 3.3% of patients. Macroscopic primary tumor specimen volume and area were available in 52.0% and 59.1% of patients, respectively, and their availability differed by period. Laboratory parameters and dermal mitotic rate showed variable missingness (particularly dermal mitotic rate), and corresponding findings were interpreted as exploratory. In addition, macroscopic primary specimen dimensions were frequently missing because the primary diagnosis was established externally in most cases and the originating pathology report had to be requested; thus, availability increased over time and may differ by center. Accordingly, analyses of primary specimen area and volume were treated as exploratory proxies and interpreted with caution.

## 3. Results

### 3.1. Study Population

A total of 2960 patients were included: 1162 in Phase 1, 1251 in Phase 2, and 547 in Phase 3. Baseline characteristics by period are shown in Table 1. Median age at diagnosis was 66 years (IQR 53–77) in Phase 1, 66 (55–79) in Phase 2, and 65 (54–78) in Phase 3 (*p* = 0.205). The proportion of male patients was similar across periods (47.8%, 49.8%, and 49.5%; *p* = 0.579).

### 3.2. Primary Outcome: Breslow Tumor Thickness

Among invasive melanomas, median TT was 1.1 mm (IQR 0.6–2.3) in Phase 1, 1.1 mm (0.5–2.4) in Phase 2, and 1.0 mm (0.5–2.3) in Phase 3 (Kruskal–Wallis *p* = 0.037). In the adjusted log-linear regression model, there was no evidence of an increase in tumor thickness during the pandemic or post-pandemic period. Compared with Phase 1, the adjusted geometric mean ratio was 0.97 (95% CI 0.90–1.04; *p* = 0.377) for Phase 2 and 0.94 (0.86–1.03; *p* = 0.167) for Phase 3 (Table 2), corresponding to approximately 3% and 6% lower adjusted mean TT, respectively, with confidence intervals compatible with no meaningful change. Center effects were substantial; for example, Dortmund showed a 24% higher adjusted mean TT than Bochum (GMR 1.24, 95% CI 1.15–1.33).

### 3.3. Center Heterogeneity (Primary Endpoint)

In the invasive melanoma TT model, the period-by-center interaction was statistically significant (*p* = 0.006), indicating heterogeneity across centers. Center-stratified models suggested that the lower post-pandemic thickness in Bochum (Phase 3 vs. Phase 1 ratio 0.82, 95% CI 0.72–0.93; *p* = 0.002), corresponding to an 18% lower adjusted mean TT, drove the interaction, whereas no significant changes were observed in Dortmund or Unna (Table S1).

### 3.4. T Categories and AJCC Stage

**Figure 1:**
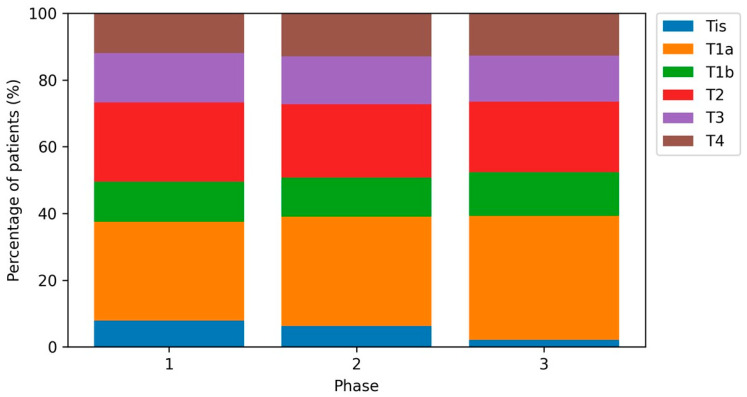
Distribution of T categories (Tis, T1a, T1b, T2, T3, T4) by study period. Percentages refer to the total cohort within each period. Pearson’s chi-squared test *p* = 0.001.

**Figure 2:**
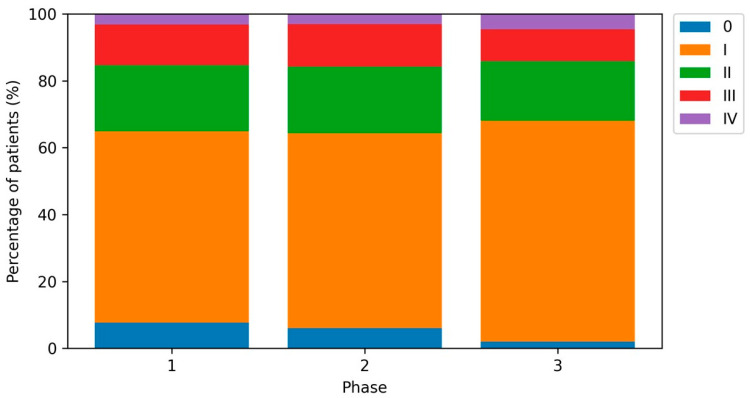
Distribution of AJCC stages (0–IV) by study period. Pearson’s chi-squared test *p* < 0.001.

The distribution of T categories differed across periods (*p* = 0.001), largely driven by a decrease in in situ lesions (Tis: 7.7% in Phase 1, 6.1% in Phase 2, and 2.0% in Phase 3; Figure 1). When restricting to invasive tumors only (T1a–T4), the T-category distribution did not differ significantly by period (*p* = 0.449). AJCC stage distribution across all stages (0–IV) also differed significantly (*p* < 0.001), again primarily due to fewer stage 0 melanomas in Phase 3 (Figure 2). After excluding stage 0, AJCC stages I–IV did not differ significantly across periods (*p* = 0.110; Figure S2). After exclusion of melanoma in situ cases (Tis), the distribution of invasive T categories (T1a–T4) did not differ significantly by period (*p* = 0.449; Figure S3).

In multivariable logistic regression, the odds of being diagnosed with stage 0 melanoma were lower in Phase 2 compared with Phase 1 (OR 0.76, 95% CI 0.55–1.05; *p* = 0.097) and markedly lower in Phase 3 (OR 0.24, 0.13–0.46; *p* < 0.001) (Table 3). Among patients with stages I–IV, there was no evidence of a shift toward advanced stages (III–IV vs. I–II) during the pandemic or post-pandemic periods (Table 3).

### 3.5. Macroscopic Primary Tumor Specimen Dimensions

Primary tumor specimen volume was available for 563/1162 (48.4%) patients in Phase 1, 665/1251 (53.2%) in Phase 2, and 310/547 (56.7%) in Phase 3 (*p* = 0.003). Among available cases, median volume increased from 1000 mm^3^ (IQR 122–3845) in Phase 1 to 1323 mm^3^ (220–4560) in Phase 2 and remained elevated in Phase 3 (1396 mm^3^, 354–3609; *p* = 0.042) (Table 4). In models addressing availability and adjusted period effects among invasive melanomas with TT and volume available, the period effect on volume was attenuated and not statistically significant (Table S2). Primary tumor specimen area was available for 638/1162 (54.9%) patients in Phase 1, 759/1251 (60.7%) in Phase 2, and 353/547 (64.5%) in Phase 3 (*p* < 0.001). Median area increased from 180 mm^2^ (IQR 48–479) in Phase 1 to 220 mm^2^ (60–519) in Phase 2 and remained elevated in Phase 3 (216 mm^2^, 75–540; *p* = 0.030, Table 4).

Pairwise comparisons suggested higher area in Phase 2 and Phase 3 versus Phase 1; after Bonferroni correction, the Phase 1 vs. Phase 3 comparison remained statistically significant (adjusted *p* = 0.049). In models addressing availability and adjusted period effects among invasive melanomas with TT and area available, the period effect on area was attenuated and not statistically significant (Table S3).

### 3.6. Laboratory Parameters and Dermal Mitotic Rate (Exploratory)

LDH, S100 and CRP values were available for 2777, 2740 and 2907 patients, respectively, and dermal mitotic rate for 1014 patients. Median LDH increased slightly across periods (Table 5).

The proportion of elevated LDH increased from 28.1% in Phase 1 to 32.3% in Phase 2 and 37.3% in Phase 3 (*p* < 0.001). Elevated S100 decreased during Phase 2 (9.9%) compared with Phase 1 (16.5%) and partially rebounded in Phase 3 (12.3%) (*p* < 0.001). CRP showed no clear period-related shift (*p* = 0.351 for elevated CRP). Dermal mitotic rate (≥1 vs. 0) did not differ significantly across periods among available cases (*p* = 0.308; Table 5). In exploratory multivariable models among invasive melanomas, elevated LDH remained more frequent in Phase 3, whereas elevated S100 remained less frequent in Phase 2 and Phase 3 (Table S4).

### 3.7. Center-Specific Analyses of Macroscopic Dimensions

Macroscopic specimen dimensions differed substantially between centers (Table S5). Within-center comparisons indicated that temporal increases in both volume and area were largely driven by Bochum, whereas Dortmund and Unna showed no clear period-related shifts (Table S6).

## 4. Discussion

In this multicenter hospital-based cohort spanning pre-pandemic, pandemic and post-pandemic periods, we observed a marked reduction in in situ melanoma presentations in the post-pandemic period, but no clinically meaningful increase in TT among invasive melanomas after covariate adjustment. In parallel, macroscopic primary tumor specimen dimensions (area and volume) were higher during and after the pandemic among available cases, although these findings were attenuated after adjustment for TT.

These findings add a post-pandemic perspective to a literature that has largely focused on comparisons between the pre-pandemic and early pandemic phases [5,7,8,9,10,11,12,13,14,15,16,17,18,19,20]. Several large cohorts and meta-analyses reported reduced melanoma diagnoses and, in many settings, thicker and/or more ulcerated tumors during periods of restricted access to care. For example, the French multicenter RIC-Mel study reported an increase in median TT and a shift toward less favorable features during the pandemic compared with 2019 [19]. A recent systematic review and meta-analysis found a pooled increase in Breslow thickness of approximately 0.2–0.3 mm and higher odds of ulceration during the COVID era, albeit with substantial heterogeneity across studies [20].

However, not all regions reported a clear thickness or stage shift. Two dermatologic clinics in North Rhine-Westphalia reported stable tumor thickness and AJCC stage distributions in 2020 and 2021 compared with 2019, with a simultaneous shift toward outpatient surgery and an increase in SLNB positivity [14]. An update from the same setting extending follow-up to 2023 suggested that key parameters returned to pre-pandemic levels only in 2023, highlighting the importance of post-pandemic observations [13]. Our results for TT are broadly consistent with these North Rhine-Westphalian observations and illustrate that a stable invasive thickness distribution can coexist with period-dependent changes in very early disease.

The apparent discrepancy between a substantial reduction in stage 0/Tis cases and the lack of a parallel increase in TT among invasive melanoma likely reflects a combination of case-mix and health-system factors. In addition, the tempo of progression from melanoma in situ/radial growth to invasive vertical growth is variable; therefore, a delay of around one year may not translate into uniform increases in Breslow thickness but can still reduce the detection of early lesions. First, the present cohort is restricted to patients with inpatient management at tertiary dermato-oncological centers. In situ melanomas and very thin invasive melanomas are frequently managed entirely in the outpatient setting and may be underrepresented even in pre-pandemic periods. During and after the pandemic, a further shift in low-risk cases toward outpatient pathways or a higher threshold for referral could preferentially reduce the number of Tis cases captured in hospital-based datasets without materially altering the distribution of invasive thickness among referred patients. Second, thicker and symptomatic tumors may generate a stronger impetus for patients and physicians to seek timely specialist care even under restrictions, whereas asymptomatic lesions detected through opportunistic screening may be more sensitive to interruptions in routine care. Third, pandemic-related work restrictions could have had opposing effects: fear of infection and reduced elective appointments may have increased the threshold to seek care, while reduced commuting and greater scheduling flexibility may have lowered practical barriers for some patients. The net effect could therefore differ across patient subgroups and healthcare settings. The fact that the reduction in stage 0/Tis persisted into Phase 3 supports the notion of delayed recovery or continued shifting of early detection and referral pathways.

The observation of higher macroscopic primary tumor specimen area and volume, persisting into the post-pandemic period among available cases, is intriguing and may point toward changes not captured by thickness alone, such as lateral tumor extent, surgical planning, referral thresholds, or documentation practices. Conceptually, lateral extent and thickness represent different growth dimensions and may be only weakly correlated in some settings [21,22,23,24]. Moreover, recent work has proposed that two-dimensional measures (e.g., calculated tumor area) provide complementary prognostic information beyond thickness in invasive melanoma [21,22]. In our cohort, period effects on area and volume were attenuated after adjustment for TT and covariates, which is compatible with a mixture of modest changes in lateral extent and documentation effects rather than a strong independent shift. Nonetheless, the descriptive increases, together with the persistent reduction in stage 0, support the hypothesis that delayed or altered detection pathways could allow lesions to become larger in surface extent without necessarily becoming thicker, particularly for superficial spreading melanomas in which radial growth may precede pronounced vertical growth.

An additional observation was pronounced center-to-center variation in recorded macroscopic specimen dimensions. Specimen volume and area were substantially smaller in Bochum than in Dortmund and Unna, despite geographic proximity of the catchment areas. Because most index excisions were performed in community dermatology practices and dimension data were abstracted from external pathology reports, this heterogeneity may reflect differences in referral networks and case mix, early detection pathways, outpatient excision margins, and/or reporting conventions across pathology laboratories rather than true differences in biological tumor size. While earlier detection (and therefore smaller lesions requiring smaller excisions) in the Bochum catchment is a plausible explanation, this remains hypothesis-generating and should be evaluated in future studies with standardized clinical diameter/area documentation. We must emphasize, however, that the differences in macroscopically determined tumor dimensions are largely attributable to the referring dermatologists, as in our centers usually only the re-excisions were performed, while most primary tumor excisions had been carried out externally.

In exploratory analyses, we observed an increased proportion of elevated LDH and a decreased proportion of elevated S100 across periods. Because these parameters are influenced by staging practices, comorbidities, and selective testing, and because dermal mitotic rate and some laboratory values were missing for subsets of patients, these findings should be interpreted cautiously and viewed as hypothesis-generating. Finally, our analysis confirmed heterogeneity in period effects on TT across centers, with evidence of a lower post-pandemic TT in Bochum but no statistically significant changes in Dortmund or Unna. Such heterogeneity may reflect center-specific referral pathways, catchment dynamics, or differences in patient selection and documentation, underscoring the need to account for center in multicenter hospital-based analyses.

This study has limitations inherent to its retrospective design. The cohort is hospital-based and limited to patients with an inpatient episode, introducing selection bias and limiting inference to population-level incidence. In the participating centers, inpatient management usually reflects planned wide local excision, sentinel lymph node biopsy and/or staging work-up; conversely, many melanoma in situ and very thin invasive melanomas are treated entirely in outpatient dermatology practices and may never be referred for inpatient care. Accordingly, outpatient diagnoses and excisions performed entirely in ambulatory practices are under-represented and may partly explain the low overall proportion of stage 0 cases and the pronounced post-pandemic drop observed in our dataset; changes in referral thresholds or inpatient capacity could further amplify this effect. Information on referral source (e.g., outpatient dermatologist vs. other) was not captured as a structured variable, although in routine care the index diagnostic excision of the primary melanoma is commonly performed in outpatient practices before referral for subsequent procedures at the study centers. Macroscopic primary tumor specimen dimensions were derived from routine pathology documentation, were missing in a substantial proportion of patients, and their availability differed by period, which may bias comparisons. Laboratory parameters and dermal mitotic rate were not available for all patients and may reflect selective testing and documentation. Our time structure was based on three broad periods and did not model finer temporal changes within phases (e.g., specific lockdown intervals). Residual confounding by unmeasured factors (e.g., referral pathways, access to screening, socioeconomic status) cannot be excluded. In addition, because most primary melanomas were excised in community dermatology practices prior to referral and dimensions were extracted from external pathology reports, the recorded measures may capture excision specimen size and margin practice rather than standardized clinical tumor diameter. Differences between centers may therefore reflect heterogeneity in outpatient excision and reporting conventions. These findings should be considered hypothesis-generating. Finally, missing data represent an important limitation of this retrospective multicenter study. Several variables, particularly macroscopic primary tumor specimen dimensions and dermal mitotic rate, were incompletely documented due to heterogeneous reporting practices across centers and referring outpatient pathology laboratories, and missingness is therefore unlikely to be completely at random. Accordingly, complete-case analyses were performed, and findings from secondary and exploratory analyses should be interpreted with caution.

## 5. Conclusions

Despite the large body of prior work on COVID-19-related disruptions in melanoma care [12,25,26,27,28,29,30,31,32,33,34,35,36,37,38,39,40,41,42,43,44,45,46,47,48,49,50,51,52,53,54,55,56,57,58,59], our multicenter data add complementary evidence from a hospital-based cohort spanning the pre-pandemic, pandemic, and post-pandemic periods. In three North Rhine-Westphalian dermato-oncology centers, the post-pandemic period was characterized by fewer in situ melanoma presentations, while TT among invasive melanomas did not increase after adjustment for case-mix. These findings suggest that pandemic-related disruptions may have affected early detection and low-risk pathways more than the presentation of invasive disease in a tertiary-care cohort. Integrating hospital data with outpatient networks and standardized measures of lesion size could further clarify post-pandemic recovery patterns and guide strategies to maintain melanoma screening and referral pathways during future healthcare disruptions.

## Figures and Tables

**Table 1:** Patient and tumor characteristics by study period.

Characteristic	Phase 1 (Pre-Pandemic)	Phase 2 (Pandemic)	Phase 3 (Post-Pandemic)	*p* Value
Patients, n	1162	1251	547	
Age at diagnosis, years, median (IQR)	66 (53–77)	66 (55–79)	65 (54–78)	0.205
Male sex, n (%)	555 (47.8%)	623 (49.8%)	271 (49.5%)	0.579
Center				0.448
Bochum, n (%)	640 (55.1%)	706 (56.4%)	300 (54.8%)	
Dortmund, n (%)	361 (31.1%)	358 (28.6%)	156 (28.5%)	
Unna, n (%)	161 (13.9%)	187 (14.9%)	91 (16.6%)	
Anatomical site				0.461
Head/neck, n (%)	205 (17.6%)	243 (19.4%)	102 (18.6%)	
Trunk, n (%)	358 (30.8%)	394 (31.5%)	183 (33.5%)	
Upper extremity, n (%)	241 (20.7%)	220 (17.6%)	111 (20.3%)	
Lower extremity, n (%)	319 (27.5%)	349 (27.9%)	137 (25.0%)	
Other, n (%)	39 (3.4%)	45 (3.6%)	14 (2.6%)	
Histologic subtype				0.504
Other/unspecified, n (%)	182 (15.7%)	186 (14.9%)	87 (15.9%)	
Superficial spreading, n (%)	645 (55.5%)	684 (54.7%)	308 (56.3%)	
Lentigo maligna, n (%)	114 (9.8%)	146 (11.7%)	62 (11.3%)	
Acral lentiginous, n (%)	46 (4.0%)	40 (3.2%)	11 (2.0%)	
Nodular, n (%)	175 (15.1%)	195 (15.6%)	79 (14.4%)	
Breslow thickness (invasive melanoma), mm, median (IQR)	1.1 (0.6–2.3)	1.1 (0.5–2.4)	1.0 (0.5–2.3)	0.037
Ulceration present, n (%)	215 (18.5%)	242 (19.3%)	101 (18.5%)	0.842
T category				0.001
Tis, n (%)	89 (7.7%)	76 (6.1%)	11 (2.0%)	
T1a, n (%)	335 (28.8%)	396 (31.7%)	199 (36.4%)	
T1b, n (%)	136 (11.7%)	142 (11.4%)	70 (12.8%)	
T2, n (%)	269 (23.1%)	267 (21.3%)	113 (20.7%)	
T3, n (%)	168 (14.5%)	174 (13.9%)	74 (13.5%)	
T4, n (%)	135 (11.6%)	156 (12.5%)	68 (12.4%)	
AJCC stage (grouped)				<0.001
Stage 0, n (%)	89 (7.7%)	76 (6.1%)	11 (2.0%)	
Stage I, n (%)	665 (57.2%)	729 (58.3%)	361 (66.0%)	
Stage II, n (%)	230 (19.8%)	249 (19.9%)	98 (17.9%)	
Stage III, n (%)	141 (12.1%)	158 (12.6%)	52 (9.5%)	
Stage IV, n (%)	37 (3.2%)	39 (3.1%)	25 (4.6%)	
Lymph node ultrasound performed, n (%)	764 (65.7%)	849 (67.9%)	423 (77.3%)	<0.001
Extended staging performed, n (%)	509 (43.8%)	503 (40.2%)	152 (27.8%)	<0.001
Sentinel lymph node biopsy performed, n (%)	588 (50.6%)	601 (48.0%)	243 (44.4%)	0.055
Positive sentinel lymph node (among informative SLNB), n (%)	122 (21.1%)	113 (19.0%)	39 (17.0%)	0.379
Primary tumor specimen volume (mm^3^), median (IQR) [available cases]	1000 (122–3845) (563)	1323 (220–4560) (665)	1396 (354–3609) (310)	0.042

**Table 2:** Multivariable log-linear regression for Breslow tumor thickness among invasive melanoma (geometric mean ratios).

Predictor	Geometric Mean Ratio (95% CI)	*p* Value
Phase 2 vs. Phase 1	0.97 (0.90–1.04)	0.377
Phase 3 vs. Phase 1	0.94 (0.86–1.03)	0.167
Age (per 10 years)	1.11 (1.09–1.13)	<0.001
Male vs. female	1.09 (1.02–1.17)	0.009
Center Dortmund vs. Bochum	1.24 (1.15–1.33)	<0.001
Center Unna vs. Bochum	1.06 (0.96–1.18)	0.266
Trunk vs. head/neck	1.12 (1.00–1.27)	0.051
Upper extremity vs. head/neck	1.06 (0.93–1.20)	0.392
Lower extremity vs. head/neck	1.05 (0.93–1.18)	0.474
Other site vs. head/neck	2.76 (1.68–4.54)	<0.001
Superficial spreading vs. other/unspecified	0.51 (0.46–0.56)	<0.001
Lentigo maligna vs. other/unspecified	0.24 (0.21–0.29)	<0.001
Acral lentiginous vs. other/unspecified	0.80 (0.63–1.03)	0.088
Nodular vs. other/unspecified	1.86 (1.66–2.10)	<0.001

**Table 3:** Multivariable logistic regression models for in situ melanoma (AJCC stage 0) and advanced stage outcomes (adjusted odds ratios).

Outcome	Contrast	Adjusted OR (95% CI)	*p* Value
In situ melanoma (AJCC stage 0)	Phase 2 vs. Phase 1	0.76 (0.55–1.05)	0.097
In situ melanoma (AJCC stage 0)	Phase 3 vs. Phase 1	0.24 (0.13–0.46)	<0.001
Advanced stage (AJCC III–IV among I–IV)	Phase 2 vs. Phase 1	1.00 (0.80–1.25)	0.980
Advanced stage (AJCC III–IV among I–IV)	Phase 3 vs. Phase 1	0.84 (0.63–1.13)	0.246
Stage IV (AJCC IV among I–IV)	Phase 2 vs. Phase 1	0.93 (0.59–1.47)	0.758
Stage IV (AJCC IV among I–IV)	Phase 3 vs. Phase 1	1.36 (0.81–2.29)	0.248

**Table 4:** Macroscopic primary tumor specimen dimensions by study period (available cases).

Parameter	Phase 1 (Pre-Pandemic)	Phase 2 (Pandemic)	Phase 3 (Post-Pandemic)	*p* Value
Primary tumor specimen area (mm^2^), median (IQR) (available cases)	180 (48–479) (638)	220 (60–519) (759)	216 (75–540) (353)	0.030
Primary tumor specimen volume (mm^3^), median (IQR) (available cases)	1000 (122–3845) (563)	1323 (220–4560) (665)	1396 (354–3609) (310)	0.042

**Table 5:** Laboratory parameters and dermal mitotic rate by study period (available cases).

Parameter	Phase 1 (Pre-Pandemic)	Phase 2 (Pandemic)	Phase 3 (Post-Pandemic)	*p* Value
LDH (U/L), median (IQR) (available cases)	191 (170–219) (1076)	196 (173–223) (1184)	202 (177–228) (517)	<0.001
LDH elevated (≥215 U/L), n/N (%)	302/1076 (28.1%)	382/1184 (32.3%)	193/517 (37.3%)	<0.001
S100 (µg/L), median (IQR) (available cases)	0.060 (0.042–0.090) (1086)	0.057 (0.036–0.075) (1149)	0.058 (0.040–0.081) (505)	<0.001
S100 elevated (≥0.11 µg/L), n/N (%)	179/1086 (16.5%)	114/1149 (9.9%)	62/505 (12.3%)	<0.001
CRP (mg/L), median (IQR) (available cases)	3.0 (3.0–3.0) (1134)	3.0 (3.0–3.0) (1229)	3.0 (3.0–3.0) (545)	0.318
CRP elevated (≥5 mg/L), n/N (%)	209/1134 (18.4%)	201/1228 (16.4%)	89/545 (16.3%)	0.351
Dermal mitotic rate ≥ 1, n/N (%)	133/372 (35.8%)	134/341 (39.3%)	101/301 (33.6%)	0.308

## Data Availability

The data that support the findings of this study are available from the corresponding author upon reasonable request.

## Supplementary Materials

The following supporting information can be downloaded at: https://www.mdpi.com/article/10.3390/cancers18030539/s1, Table S1: Center-stratified period effects on Breslow tumor thickness among invasive melanoma (geometric mean ratios); Table S2: Primary tumor specimen volume availability and multivariable models for primary tumor specimen volume (ratios/odds ratios); Table S3: Primary tumor specimen area availability and multivariable models for primary tumor specimen area (ratios/odds ratios); Table S4: Exploratory multivariable models for elevated laboratory parameters and dermal mitotic rate among invasive melanomas; Table S5: Macroscopic primary tumor specimen dimensions by center (available cases); Table S6: Macroscopic primary tumor specimen dimensions by center and study period (available cases); Figure S1: Distribution of Breslow tumor thickness (TT) among invasive melanomas. The left panel shows the right-skewed distribution of TT in millimeters. The right panel shows the distribution after logarithmic transformation, demonstrating reduced skewness and supporting the use of log-linear regression models in the analysis; Figure S2: Distribution of AJCC stages I–IV (excluding stage 0) by study period. Pearson’s chi-squared test P = 0.110; Figure S3: Sensitivity analysis: distribution of invasive T categories (T1a–T4) by study period after exclusion of melanoma in situ (Tis). Percentages refer to the invasive cohort within each period. Pearson’s chi-squared test P = 0.449.
