# Supplementary material for: Melanoma Presentations Before, During, and After the COVID-19 Pandemic: A Multicenter Cohort Study from North Rhine-Westphalia, Germany
Source: Cancers (Basel). 2026 Feb 6;18(3):539. doi: 10.3390/cancers18030539 (PMC12896566; doi:10.3390/cancers18030539)
Supplement: Supplementary file 1 [file cancers-18-00539-s001.zip › cancers-4100205-supplementary.pdf]

Supplementary material

Supplementary Tables

Supplementary Table S1. Center-stratified period effects on Breslow tumor thickness among invasive melanoma (geometric mean ratios).

Center	Contrast	Geometric mean ratio (95% CI)	P value
Bochum	Phase 2 vs Phase 1	0.95 (0.86–1.05)	0.314
Bochum	Phase 3 vs Phase 1	0.82 (0.72–0.93)	0.002
Dortmund	Phase 2 vs Phase 1	0.99 (0.88–1.11)	0.834
Dortmund	Phase 3 vs Phase 1	1.10 (0.95–1.26)	0.202
Unna	Phase 2 vs Phase 1	1.04 (0.85–1.26)	0.709
Unna	Phase 3 vs Phase 1	1.18 (0.92–1.52)	0.186

Supplementary Table S2. Primary tumor specimen volume availability and multivariable models for primary tumor specimen volume (ratios/odds ratios).

Outcome/Model	Contrast	Ratio/OR (95% CI)	P value
Primary tumor specimen volume recorded	Phase 2 vs Phase 1	1.21 (1.03–1.41)	0.022
Primary tumor specimen volume recorded	Phase 3 vs Phase 1	1.39 (1.13–1.70)	0.002
Primary tumor specimen volume (log-linear model, adjusted)	Phase 2 vs Phase 1	1.10 (0.90–1.34)	0.366
Primary tumor specimen volume (log-linear model, adjusted)	Phase 3 vs Phase 1	1.16 (0.92–1.47)	0.214

Supplementary Table S3. Primary tumor specimen area availability and multivariable models for primary tumor specimen area (ratios/odds ratios).

Outcome/Model	Contrast	Ratio/OR (95% CI)	P value
Primary tumor specimen area recorded	Phase 2 vs Phase 1	1.27 (1.08–1.49)	0.004
Primary tumor specimen area recorded	Phase 3 vs Phase 1	1.49 (1.21–1.84)	<0.001
Primary tumor specimen area (log-linear model, adjusted)	Phase 2 vs Phase 1	1.06 (0.92–1.21)	0.446

Primary tumor specimen area (log-linear model, adjusted)	Phase 3 vs Phase 1	1.10 (0.94–1.29)	0.250

Supplementary Table S4. Exploratory multivariable models for elevated laboratory parameters and dermal mitotic rate among invasive melanomas.

Outcome	Contrast	Adjusted OR (95% CI)	P value
Elevated LDH (>=215 U/L)	Phase 2 vs Phase 1	1.26 (1.03–1.54)	0.023
Elevated LDH (>=215 U/L)	Phase 3 vs Phase 1	1.74 (1.36–2.22)	<0.001
Elevated S100 (>=0.11 µg/L)	Phase 2 vs Phase 1	0.50 (0.37–0.65)	<0.001
Elevated S100 (>=0.11 µg/L)	Phase 3 vs Phase 1	0.63 (0.45–0.89)	0.009
Elevated CRP (>=5 mg/L)	Phase 2 vs Phase 1	0.81 (0.65–1.02)	0.080
Elevated CRP (>=5 mg/L)	Phase 3 vs Phase 1	0.84 (0.63–1.13)	0.250
Dermal mitotic rate >=1	Phase 2 vs Phase 1	0.95 (0.64–1.41)	0.791
Dermal mitotic rate >=1	Phase 3 vs Phase 1	0.70 (0.45–1.07)	0.100

Supplementary Table S5. Macroscopic primary tumor specimen dimensions by center (available cases).

Center	Specimen volume, median (IQR) [n]	Specimen area, median (IQR) [n]	P value across periods (volume)	P value across periods (area)
Bochum	640 (96–2500) [858]	143 (36–360) [989]	0.003	0.007
Dortmund	2400 (665–8140) [445]	350 (128–774) [501]	0.256	0.568
Unna	2025 (540–5145) [235]	312 (100–612) [260]	0.858	0.868

Supplementary Table S6. Macroscopic primary tumor specimen dimensions by center and study period (available cases).

Center	Phase	Specimen volume, median (IQR) [n]	Specimen area, median (IQR) [n]
Bochum	1	392 (64–2275) [353]	108 (28–324) [399]
Bochum	2	759 (150–2515) [354]	150 (45–375) [413]
Bochum	3	900 (155–2828) [151]	170 (49–405) [177]
Dortmund	1	2580 (850–8944) [158]	375 (152–870) [175]
Dortmund	2	2415 (450–9828) [193]	356 (116–800) [218]
Dortmund	3	2130 (626–4590) [94]	311 (143–626) [108]

Unna	1	1860 (669–5998) [52]	260 (103–514) [64]
Unna	2	2132 (667–5030) [118]	343 (98–634) [128]
Unna	3	2025 (360–4800) [65]	288 (101–580) [68]

Supplementary Figure

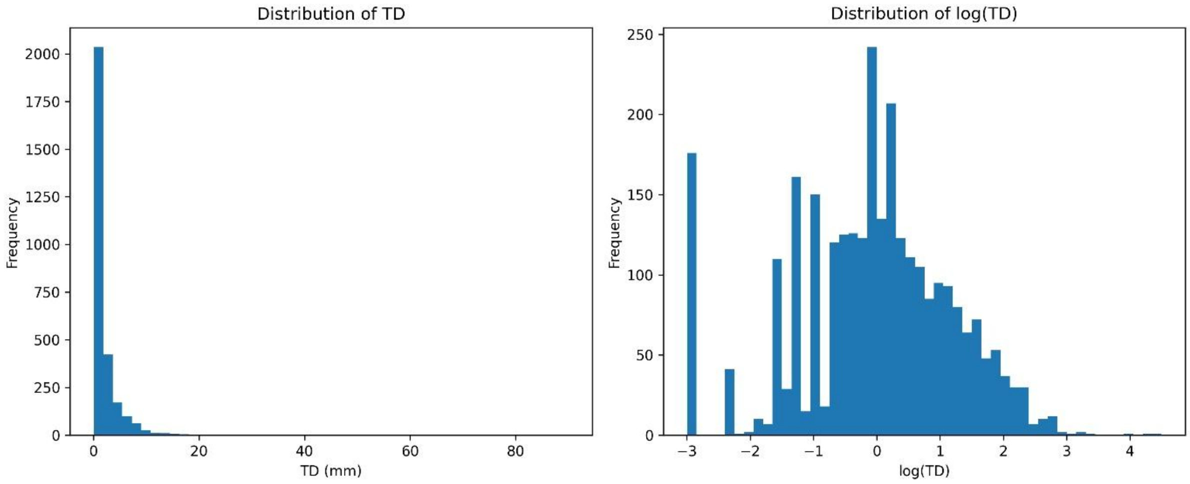

Supplementary Figure S1. Distribution of tumor diameter (TD) across all phases and centers and of log-transformed TD (histograms), illustrating right-skewness and the rationale for log transformation.

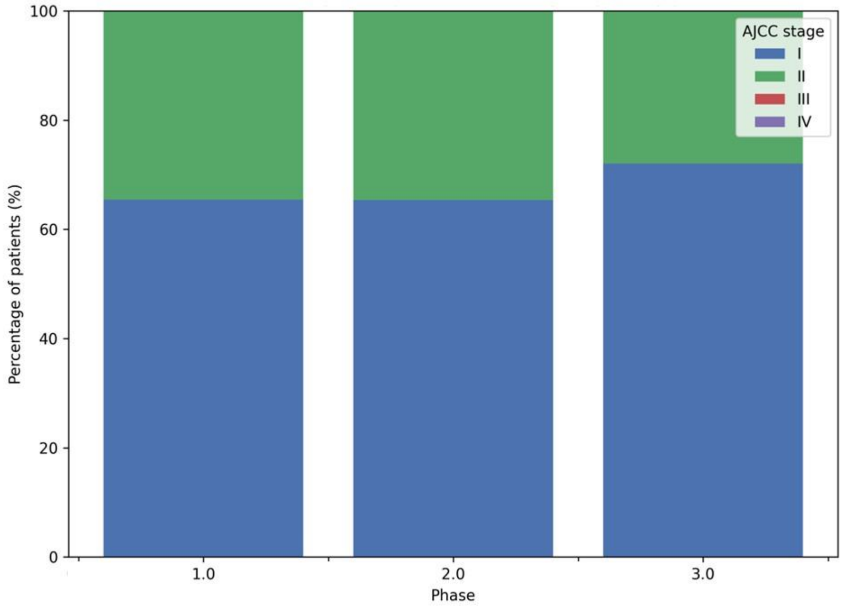

Supplementary Figure S2. Distribution of AJCC stages I–IV (excluding stage 0) by study period. Pearson's chi-squared test P=0.110.

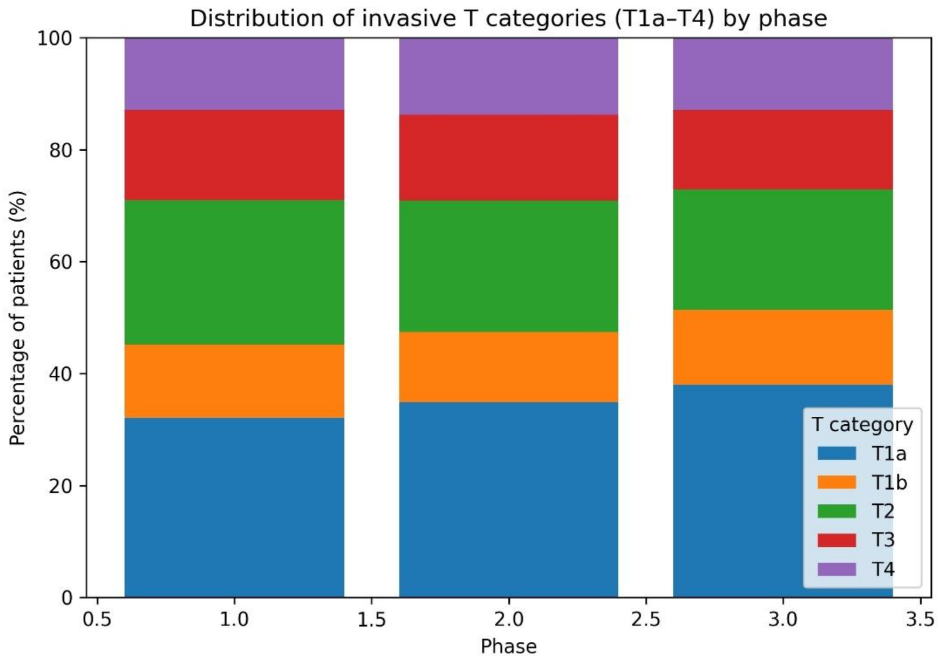

Supplementary Figure S3. Sensitivity analysis: distribution of invasive T categories (T1a–T4) by study period after exclusion of melanoma in situ (Tis). Percentages refer to the invasive cohort within each period. Pearson's chi-squared test P=0.449.
